# Early changes of microRNAs in blood one month after bariatric surgery

**DOI:** 10.1186/s13098-024-01364-2

**Published:** 2024-07-15

**Authors:** Guanhua Lu, Huanhuan Gao, Ruixiang Hu, Ji Miao, Zhiyong Dong, Cunchuan Wang, Xinxin Chen

**Affiliations:** 1 Department of Breast Surgery, The Second Affiliated Hospital of Guangzhou Medical University, No. 250, Changgang East Road, Haizhu District, Guangzhou, Guangdong Province China; 2 Department of Metabolic and Bariatric Surgery, The First Affiliated Hospital of Jinan University, No. 613, Huangpu Avenue West, Tianhe District, Guangzhou, Guangdong Province China; 3 Department of Ophthalmology, The Third Affiliated Hospital of Chongqing Medical University, Chongqing, China; 4 Division of Endocrinology, Boston Children’s Hospital, Harvard Medical School, Boston, MA USA

**Keywords:** MicroRNAs, Obesity, Bariatric surgery, Sleeve gastrectomy, Roux-en-Y gastric bypass

## Abstract

**Background:**

Changes in microRNAs (miRNAs) are relevant to bariatric surgery and its comorbidities. The characteristics of changes in miRNAs of the early postoperative period following both bariatric procedures, sleeve gastrectomy (SG) and Roux-en-Y gastric bypass (RYGB), as well as the factors that related to the effectiveness of early weight loss remain unclear.

**Methods:**

We recruited 18 patients who performed SG and 15 patients who performed RYGB. Their preoperative and 1-month postoperative clinical data and fasting serum samples were collected, and the latter were analyzed by RNA-sequencing. Differential expression analysis of miRNAs was performed by the R-tool. Functional classification annotation and pathway enrichment analysis of targeted genes were analyzed by KOBAS software. The change profiles of miRNAs for both surgeries and their correlation with clinical characteristics and weight loss effectiveness were further analyzed.

**Results:**

A total of 85 differentially expressed miRNAs were identified before and after SG, while a total of 76 were found before and after RYGB. The target genes of these miRNAs were similar in the Gene Ontology enrichment analysis in SG and RYGB, and the enrichment analysis in the Kyoto Encyclopedia of Genes and Genomes was mainly related to metabolic pathways. Hsa-miR-493-5p, hsa-miR-184, and hsa-miR-3199 exhibited similar changes in SG and RYGB, and the former two were correlated with clinical characteristics. Hsa-miR-6729-5p, hsa-miR-4659b-5p, and hsa-miR-2277-5p were correlated with the weight loss effectiveness of SG, while hsa-miR-4662a-5p was correlated with the weight loss effectiveness of RYGB.

**Conclusions:**

Short-term metabolic improvement and weight loss occurring after SG and RYGB surgery might be related to changes in miRNAs, which act on multiple biological pathways by regulating genes. In addition, some clinical characteristics and miRNAs were related to the effectiveness of early weight loss after SG and RYGB surgery.

**Clinical Trial Registration:**

ChiCTR2200058333.

**Supplementary Information:**

The online version contains supplementary material available at 10.1186/s13098-024-01364-2.

## Introduction

Obesity prevalence has risen steadily in recent decades and has become a common health concern worldwide [1]. Obesity may cause a range of obesity-related complications, leading to serious health problems as well as economic burdens [2, 3]. For patients with obesity, bariatric surgery is considered the most efficient intervention and has received excellent promotion worldwide. With the maturity of bariatric surgery and the development of laparoscopic techniques, different bariatric procedures emerge in an endless stream [4, 5]. Among them, sleeve gastrectomy (SG) and Roux-en-Y gastric bypass (RYGB) are widely favored by surgeons and have become the most popular two types of bariatric surgery for their simplistic operation, positive weight loss effect, improvement of obesity-related complications, and fewer surgical complications [6–8]. Confusingly, obesity-related complications such as blood glucose metabolism, insulin resistance, hypertension, and fatty liver disease improved significantly within a short time following bariatric surgery, even though there was no notable weight loss [9, 10]. In addition, short-term weight loss outcomes are related to preoperative status and vary from person to person [11, 12]. Thence, characterizing early changes in bariatric surgery and exploring predictors of efficacy in bariatric surgery remain extremely challenging problems.

MicroRNAs (miRNAs) are about 22 nucleotides in length and are endogenous short single-stranded non-protein-coding RNAs that are highly conserved across species [13, 14]. Although miRNAs do not directly encode proteins, they are crucial to the post-transcriptional control of gene expression by fully or partially complementary binding to the 3’ untranslated region of target messenger RNAs (mRNA) [15, 16]. MiRNAs have been shown to regulate lipid metabolism, glucose metabolism, insulin secretion, and other obesity-related biological pathways, therefore finding key miRNAs by RNA-sequencing may uncover the mechanisms involved in improving metabolic syndrome and its comorbidities in bariatric surgery [9, 14, 17]. Scholars have already used miRNAs as targets for the prevention and treatment of obesity. These miRNAs achieve the effect of improving metabolism through a variety of pathways, such as controlling nerves, resisting adipocyte apoptosis, promoting the activation of brown adipose tissue, and regulating glycolipid metabolism [18]. Several studies have shown differences in miRNAs before and after bariatric surgery [19, 20], suggesting the relevance of these changes in miRNAs profiles and bariatric surgery. By analyzing 17 studies on miRNAs profiles of patients before and after bariatric surgery, Gladys Langi et al. [21] found differential expression of miRNAs in various tissues after surgery, with a total of more than 200 unique miRNAs reported. These miRNA profiles may not only serve as clinical biomarkers for predicting effectiveness after bariatric surgery, but also as important factors in regulating metabolism after bariatric surgery. In addition to this, the miRNAs change profiles were significantly different in SG and RYGB, and this difference may explain the difference in weight loss success between RYGB and SG, and more comparative studies between RYGB and SG patients are needed to confirm these observations. Furthermore, few studies have evaluated early changes in miRNAs following bariatric surgery [22, 23], and these changes may explain early weight loss and improvement in obesity-related complications.

In light of the potential association of miRNAs in bariatric surgery, this study investigated the characteristics of early changes in miRNAs 1 month after two types of surgical procedures, SG and RYGB, and performed a comparative analysis to reveal possible profiles for improving poor metabolism at an early stage after bariatric surgery. In addition, the relationship between clinical characteristics and differential miRNAs of the two surgical procedures was further analyzed, as well as exploring their potential as predictive factors in evaluating the early effectiveness of bariatric surgery.

## Methods

### Participants

From November 2020 to August 2021, patients who came to our hospital for bariatric surgery were recruited for this study. Inclusion criteria were age 20 to 50 years, any gender, body mass index (BMI) ≥ 27.5 kg/m^2^, clinical data, and blood samples were available before and 1 month after surgery. For patients who were taking medication before surgery, we recommended that they stop taking medication for 1 month after surgery for observation. If they have any discomfort or must take medication, they should consult a professional who will decide whether to continue taking medication after a comprehensive assessment. The exclusion criteria were patients with active cancer, pregnant or breastfeeding, loss of daily living ability, severe organ diseases before surgery, and severe mental or psychological diseases. As an exploratory study, a total of 18 individuals had SG and 15 had RYGB. In SG, 8 patients had comorbid type 2 diabetes mellitus and 7 had comorbid hypertension, whereas in RYGB, 10 patients had comorbid type 2 diabetes mellitus and 4 had comorbid hypertension.The same surgical team performed all of the procedures. We obtained hand-signed informed consent from each patient. This study was approved by the Institutional Review Board of our hospital.

### Anthropometric measurements

All anthropometric indices, including height, weight, neck circumference (NC), chest circumference (CC), abdominal circumference (AC), and hip circumference (HC), were obtained in a standard manner [24]. Specifically, the patient stands upright, eyes are level in front, both arms are naturally lowered, and breathing is kept steady. The values of NC, CC, AC, HC were obtained by measuring the circumference at the level of the seventh cervical vertebra behind the neck, the superior papillae, the iliac crest point, and the most prominent part of the buttocks posteriorly, respectively, using a soft tape.

### Serum samples acquisition and processing

All serum samples were kept in the Biobank of the First Affiliated Hospital of Jinan University. Patients’ blood samples were collected 1–3 days before surgery, and patients received the same dietary instructions during this time to avoid the effects of some compounds present in food on miRNAs. Patients were fasted for 12 h and their venous blood was collected by standard methods. At 4 °C, serum samples were centrifuged at 4000 g for 10 min. The supernatant was kept at -80 °C after centrifugation.

### RNA-sequencing and data analysis

Using the miRNeasy Serum/Plasma Kit (Qiagen, 217,184, Germany), total RNA was extracted from serum samples. To determine the purity, concentration, and integrity of total RNA, we assayed it by four methods, including agarose gel electrophoresis analysis, Nanodrop detector, Qubit microRNA Assay kit, and Bioptic Qsep100 analysis system. After samples detection, purification, quantification, library construction, and quality assessment, the Hiseq X-10 sequencer (Illumina, USA) was used for sequencing analysis.

Fastx_toolkit (version: 0.0.13.2) was used to filter raw sequencing data before analyzing RNA-sequencing results. To trim adaptor sequences, Cutadapt (version: 1.15) was utilized. Clean reads were further eliminated duplication and quality filtered, then mapped to Homo sapiens GRCh38. R-tool (version: 4.1.2) was used for most data analysis. The reads were mapped to the known miRNA in the miRBase (Release 22.1) database and novel miRNA was predicted using the mirdeep2 software (version: 2.0.0.8). Nextly, the target mRNA was predicted by miRanda (version: 3.3a) and RNAhybrid (version: 2.1.1). Common target genes were screened using Venn diagrams. The edgeR package (version: 3.12.1) was applied to analyze the differential expression of miRNAs, with P-value cutoff of 0.05 and Log_2_ Fold-change (Log_2_ FC) cutoff of 1 defined as statistically significant differences. Finally, for Gene Ontology (GO) and Kyoto Encyclopedia of Genes and Genomes (KEGG) enrichment analysis, a P-value < 0.05 threshold was judged statistically significant enrichment by KOBAS software (version: 2.1.1). Note that in order to compare the expression levels of known and novel miRNAs in each sample, transcripts per million reads (TPM) was used for data normalization in this study. The formula is TPM = Read count × 10^6^ / Total miRNA reads.

### Efficacy evaluation of bariatric surgery

To assess the effectiveness 1 month after bariatric surgery, we introduced percentage total weight loss (%TWL) and percentage excess weight loss (%EWL). The %TWL was calculated as follows: [(Initial weight − Postoperative weight) / Initial weight] × 100%. The %EWL referred to the following equation: [(Initial weight − Postoperative weight) / (Initial weight − Ideal weight)] × 100%. Ideal Weight was based on a BMI of 25 kg/m^2^. The %EWL ≥ 25% [25, 26] at 1 month was defined as the effective criterion of bariatric surgery, otherwise, it was ineffective.

### Statistical analysis

Data were presented as mean ± standard deviation (mean ± SD). Differential expression analysis of miRNAs was realized by R tool. To compare categorical variables, the chi-square test was employed, whereas the student’s t-test was utilized to examine continuous data. Specifically, two independent samples t-test was used to compare different surgical procedures or different effectiveness of weight loss, and paired samples t-test was used for comparison between preoperative and postoperative. Pearson correlation was used to analyze the correlation between differentially expressed miRNAs (TPM) and clinical data. A statistically significant difference is defined as P < 0.05.

## Results

### Preoperative and postoperative clinical characteristics of SG and RYGB

As shown in Table 1, 18 patients had SG and 15 had RYGB, with an average age of 30.50 years and 32.73 years, respectively. One month after bariatric surgery, there were significant changes in anthropometric parameters such as body weight, BMI, NC, CC, AC, and HC for both bariatric procedures (all P-values less than 0.05). In SG, lipid metabolism-related indicators such as total cholesterol (TCHOL), high-density lipoprotein cholesterol (HDL-C), and low-density lipoprotein cholesterol (LDL-C) did not differ before and after surgery (P > 0.05), except that triglycerides (TG) decreased significantly (P = 0.022). In contrast, in RYGB, TCHOL, HDL-C, and LDL-C decreased significantly (P < 0.05), except for TG, which was not significantly changed (P = 0.181). Glucose metabolism-related indicators such as fasting blood glucose (FBG), glycated hemoglobin (HbA1c), glycated serum protein (GSP), fasting insulin, and homeostasis model assessment of insulin resistance (HOMA-IR, calculated as fasting blood glucose × fasting insulin / 22.5) were significantly changed in both procedures, showing a trend of improvement. As the authoritative evaluation index of bariatric surgery, %TWL and %EWL showed a good weight loss effect in both procedures after one month. These results demonstrated that significant improvements in anthropometric and metabolic parameters could be observed in the short term after bariatric surgery.

**Table 1:** Baseline and 1-month postoperative clinical characteristics in patients undergoing SG or RYGB

Characteristics	SG (n = 18)			RYGB (n = 15)		
	Baseline	1 month	P-value	Baseline	1 month	P-value
Age (years)	30.50 ± 7.05	–	–	32.73 ± 8.00	–	–
Gender (M/F)	M (4); F (14)	–	–	M (8); F (7)	–	–
Weight (kg)	104.29 ± 19.30	93.54 ± 18.31	**< 0.001**	124.61 ± 27.56	111.35 ± 25.67	**< 0.001**
BMI (kg/m^2^)	38.00 ± 4.65	33.79 ± 4.51	**< 0.001**	44.74 ± 9.00	39.55 ± 8.06	**< 0.001**
NC (cm)	41.57 ± 2.97	39.29 ± 3.00	**< 0.001**	45.50 ± 4.99	43.37 ± 4.96	**< 0.001**
CC (cm)	119.21 ± 10.39	111.92 ± 8.66	**< 0.001**	131.30 ± 13.38	123.20 ± 12.04	**< 0.001**
AC (cm)	114.30 ± 12.16	106.69 ± 11.80	**< 0.001**	133.15 ± 18.38	123.10 ± 17.66	**< 0.001**
HC (cm)	119.68 ± 9.37	113.65 ± 8.58	**0.001**	131.05 ± 18.11	126.87 ± 18.25	**0.035**
WHR (ratio)	0.96 ± 0.07	0.94 ± 0.06	0.232	1.02 ± 0.07	0.97 ± 0.06	**0.029**
TCHOL (mmol/L)	5.35 ± 0.81	5.02 ± 0.92	0.190	5.43 ± 0.95	4.60 ± 0.71	**0.001**
HDL-C (mmol/L)	1.09 ± 0.20	1.03 ± 0.17	0.086	1.06 ± 0.19	0.90 ± 0.13	**0.003**
LDL-C (mmol/L)	3.24 ± 0.59	3.00 ± 0.74	0.227	3.28 ± 0.66	2.79 ± 0.58	**0.004**
TG (mmol/L)	2.02 ± 1.36	1.33 ± 0.54	**0.022**	1.79 ± 1.31	1.50 ± 0.70	0.181
FBG (mmol/L)	7.15 ± 3.86	5.01 ± 0.73	**0.017**	7.48 ± 3.51	5.56 ± 1.08	**0.027**
HbA1c (%)	6.64 ± 2.09	5.84 ± 1.20	**0.003**	7.01 ± 1.87	5.98 ± 1.24	**< 0.001**
GSP (µmol/L)	170.58 ± 68.13	136.39 ± 19.58	**0.017**	178.19 ± 85.41	136.80 ± 33.76	**0.015**
Insulin (mIU/L)	23.43 ± 13.61	11.42 ± 6.99	**0.003**	25.77 ± 13.09	13.79 ± 6.30	**0.001**
HOMA-IR	7.76 ± 5.92	2.68 ± 2.05	**0.001**	7.86 ± 3.81	3.29 ± 1.46	**0.001**
%TWL	–	10.46% ± 2.27%	–	–	10.86% ± 2.33%	–
%EWL	–	35.24% ± 17.47%	–	–	45.82% ± 70.76%	–

Data were shown as mean ± standard deviation. SG (sleeve gastrectomy); RYGB (Roux-en-Y gastric bypass); M/F (male/ female); BMI (body mass index); NC (neck circumference); CC (chest circumference); AC (abdominal circumference); HC (hip circumference); WHR (waist to hip ratio); TCHOL (total cholesterol); HDL-C (high-density lipoprotein cholesterol); LDL-C (low-density lipoprotein cholesterol); TG (triglycerides); FBG (fasting blood glucose); HbA1c (glycated hemoglobin); GSP (glycated serum protein); HOMA-IR (homeostasis model assessment of insulin resistance). %TWL (percentage total weight loss); %EWL (percentage excess weight loss). P-values < 0.05 were bolded

### Differential expression analysis of miRNAs

**Fig. 1:**
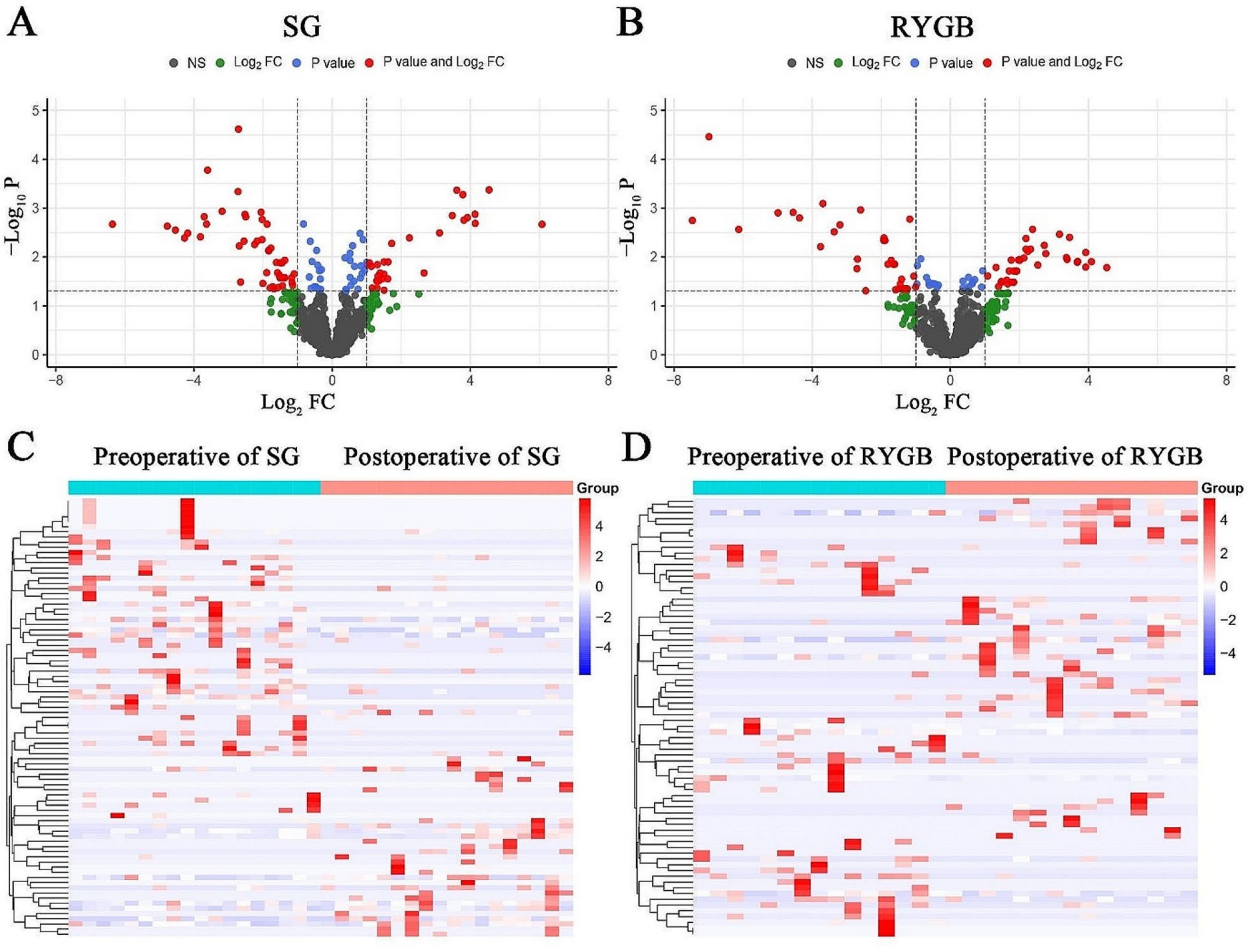
Analysis of differential expression of miRNAs. Volcano plots of differential expression of miRNAs for SG (**A**) and RYGB (**B**). Red dots indicated Log_2_ FC > 1 and P-value < 0.05, which were defined as differential expressions of miRNAs. Heatmaps of differential expression of miRNAs before and after surgery for SG (**C**) and RYGB (**D**)

We annotated known miRNAs through the miRBase database, and we also used softwares such as miREvo and mirdeep2 to predict new miRNAs, which were defined as novel miRNAs. After the normalization of miRNAs, the differentially expressed miRNAs of SG and RYGB were analyzed, including known miRNAs and novel miRNAs. A total of 1845 miRNAs were found in this study, including 1440 known miRNAs and 405 novel miRNAs. The volcano plots in Fig. 1A and B showed the distribution of differentially expressed miRNAs for the two procedures, shown as red dots. Log_2_ FC > 1 and P-value < 0.05 denoted up-regulated miRNAs, whereas Log_2_ FC < − 1 and P-value < 0.05 denoted down-regulated miRNAs. Of the miRNAs in SG, 31 were up-regulated and 54 were down-regulated (Fig. 1 and Table S1). In RYGB, there were 37 up-regulated miRNAs and 39 down-regulated miRNAs (Fig. 1 and Table S2). Further clustering analysis of the differentially expressed miRNAs revealed that the two bariatric procedures (Fig. 1C and D) had obvious changes in miRNAs before and after surgery.

### GO functional classification annotation

**Fig. 2:**
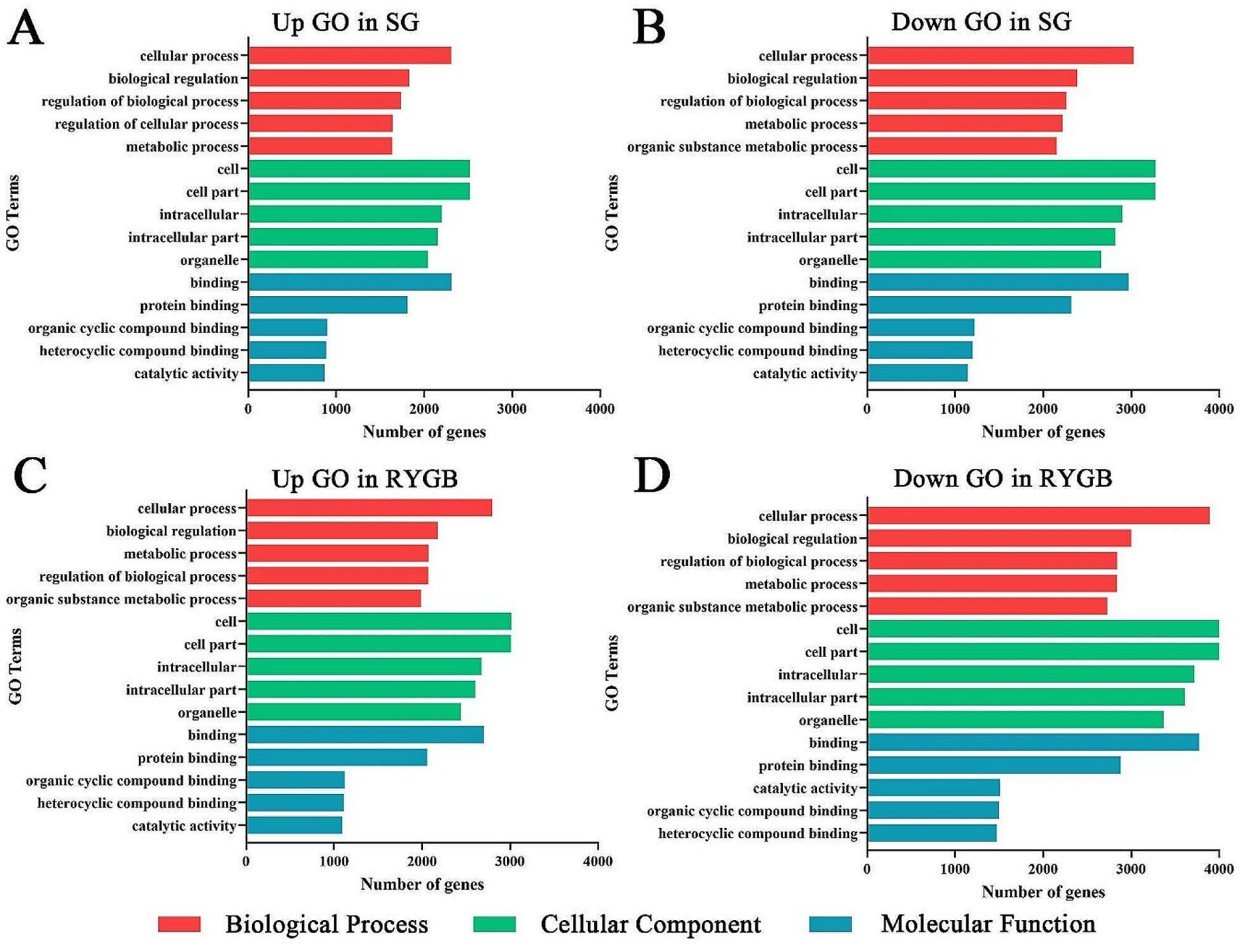
GO enrichment analysis of target genes. GO enrichment analysis of target genes predicted by up-regulated (**A**) or down-regulated miRNAs (**B**) in SG. GO enrichment analysis of target genes predicted by up-regulated (**C**) or down-regulated miRNAs (**D**) in RYGB. According to the number of genes, the top 5 GO terms in each of the three categories were selected for mapping

The miRanda and RNAhybrid databases were used to predict target genes, and the target genes prediction list used in this work was the intersection of the two databases. Using the GO database, the target genes were classified and annotated as biological process, cellular component, and molecular function, and the top 5 GO terms in each classification were displayed according to the number of genes (Fig. 2). We found that whether it was SG or RYGB, the GO terms of the target genes which were predicted by up-regulated or down-regulated miRNAs were basically the same. In biological processes, target genes were mainly enriched in cellular process, biological process, and metabolic process. While in cellular components, target genes were manifested in cell, cell part, cell interior, and organelle. Molecular functions were mainly reflected in protein binding, compound binding, and catalytic activity. The predicted target genes of these differentially expressed miRNAs might function in almost all parts of the organism.

### KEGG pathway enrichment analysis

**Fig. 3:**
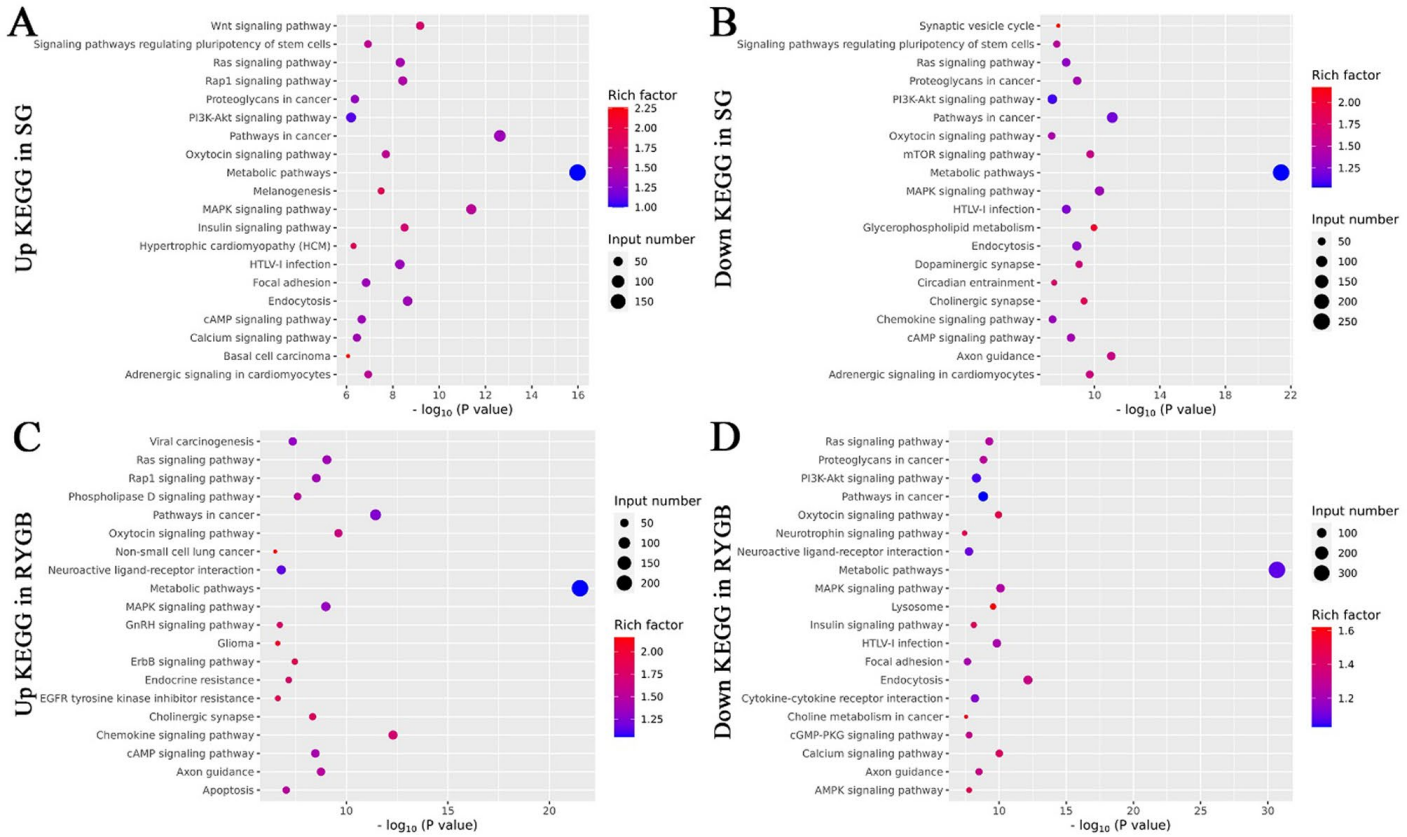
KEGG enrichment analysis of target genes. KEGG enrichment analysis of target genes predicted by up-regulated (**A**) or down-regulated miRNAs (**B**) in SG. KEGG enrichment analysis of target genes predicted by up-regulated (**C**) or down-regulated miRNAs (**D**) in RYGB. Input number referred to the number of genes enriched to each pathway. The rich factor was the ratio of the number of enriched genes to the number of background genes. The top 20 most significant KEGG terms were selected for mapping by P-value

After GO annotation, statistics were performed according to the KEGG metabolic pathways they were involved in. Figure 3 showed the top 20 most significant KEGG terms sorted by P-value. The KEGG pathway’s target gene count was represented by the size of the dots, and the degree of enrichment was shown by the rich factor. In SG, the pathways with the highest enrichment of target genes from up-regulated and down-regulated miRNAs were basal cell carcinoma (rich factor = 2.26) and synaptic vesicle cycle (rich factor = 2.17), respectively. The pathways with the largest number of target genes from up-regulated and down-regulated miRNAs both were metabolic pathways (input numbers were 191 and 252, respectively). For RYGB, the most enriched pathways in target genes of up-regulated miRNAs were maturity onset diabetes of the young (rich factor = 2.75), while the most enriched pathways in target genes of down-regulated miRNAs corresponded to butirosin and neomycin biosynthesis (rich factor = 3.18). Similar to SG, the largest number of target genes in RYGB were also metabolic pathways, with 238 target genes from up-regulated miRNAs and 338 target genes from down-regulated miRNAs. These outcomes further illustrated a possible relationship between differentially expressed miRNAs and metabolic pathways.

### Intersection of differentially expressed known miRNAs in both surgeries and their relationship to clinical characteristics

**Fig. 4:**
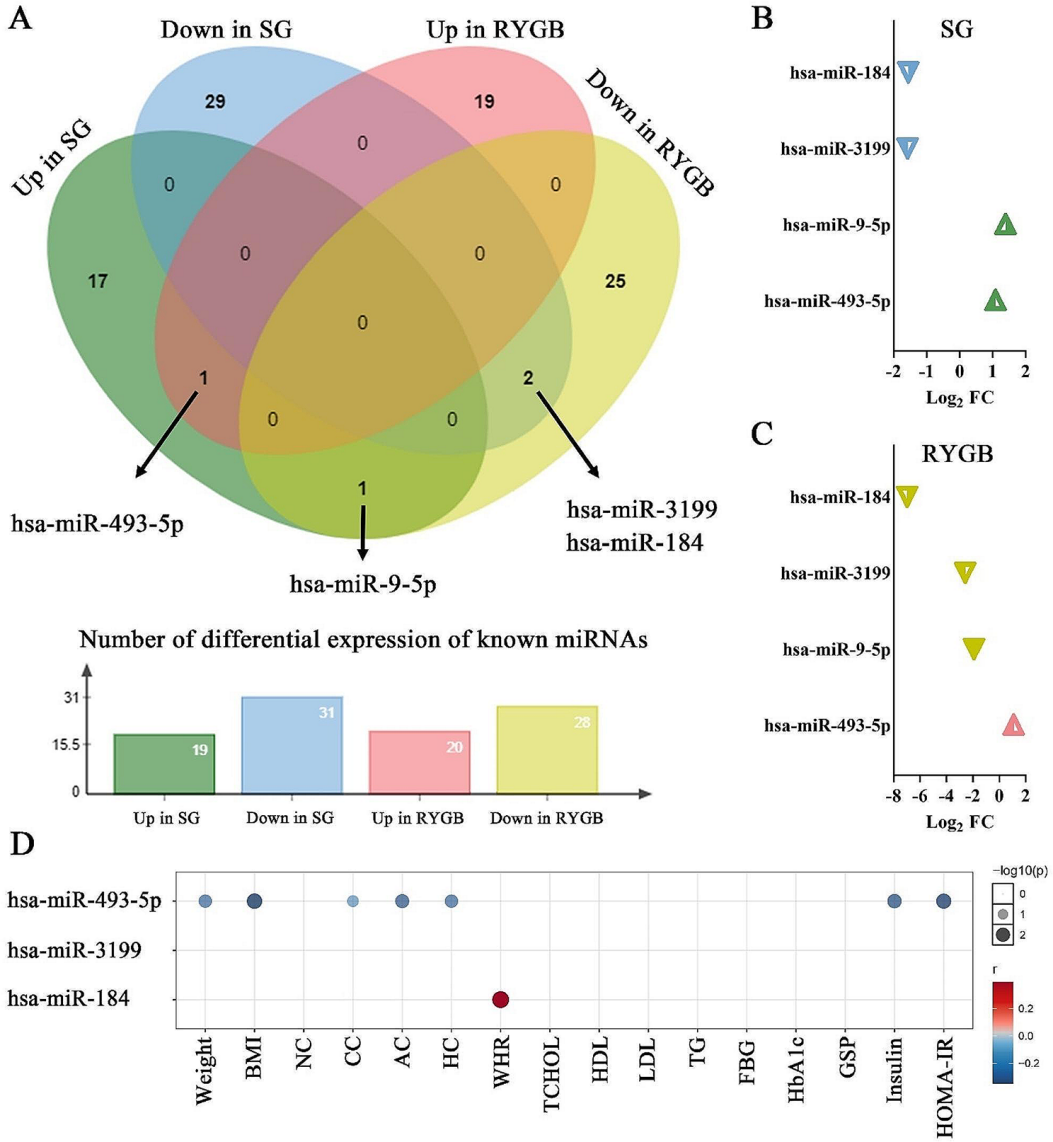
Differential expression of known miRNAs that intersected in SG and RYGB and their relationship to clinical characteristics. **A**: Venn diagram of differential expression of known miRNAs before and after both surgical procedures. **B** & **C**: The Log_2_ FC of the four intersecting differential expressions of known miRNAs in SG and RYGB, respectively. **D**: Pearson correlation of the three known miRNAs co-altered in SG and RYGB with clinical characteristics. Only values with a P-value < 0.05 were shown

Here, we only analyzed the intersection of differentially expressed known miRNAs in SG and RYGB to study their co-altered miRNAs. As shown in Fig. 4A, B, and C, in SG and RYGB, two known miRNAs were down-regulated together, which were hsa-miR-3199 and hsa-miR-184. The only known miRNAs up-regulated together in both procedures was hsa-miR-493-5p. Interestingly, hsa-miR-9-5p was up-regulated in SG, but down-regulated in RYGB. Although the number of known miRNAs differentially expressed in the two procedures was large, their intersection was rare, and these co-altered miRNAs might be the commonality of bariatric surgery.

Hsa-miR-493-5p, hsa-miR-3199, and hsa-miR-184 were simultaneously up- or down-regulated in SG and RYGB, and these three miRNAs-target genes co-occurrence networks were shown in Figure S1. We further investigated the relationship between these miRNAs with the same change profile in both procedures and clinical characteristics. As shown in Fig. 4D, there were correlations between hsa-miR-493-5p and anthropometric indices such as body weight (*r* = − 0.293), BMI(*r* = − 0.351), CC (*r* = − 0.249), AC (*r* = − 0.314), HC (*r* = − 0.295) and glucose metabolism-related indices such as insulin (*r* = − 0.321), HOMA-IR (*r* = − 0.342), indicating the negative change of hsa-miR-493-5p on these indices. That means that bariatric surgery led to a rise in hsa-miR-493-5p, which was associated with an early improvement in anthropometric indicators as well as in glucose metabolism indicators. In contrast, hsa-miR-184 only showed a positive correlation with WHR (*r* = 0.395), and presumably its downregulation in bariatric surgery was beneficial to improving WHR. Disappointingly, no correlation was found between hsa-miR-3199 and the clinical characteristics we collected.

### Evaluation of the effectiveness of weight loss at 1 month postoperatively

To evaluate the effectiveness of weight loss 1 month after the two procedures, we defined %EWL ≥ 25% as effective weight loss, otherwise, it was ineffective. In SG, we found that 13 patients were effective in weight loss, and the effective rate was 72.22%. Likewise, we found that 6 patients in RYGB were effective in weight loss, with an effective rate of 40.00% (Table 2). We divided patients’ preoperative characteristics and differentially expressed miRNAs into effective and ineffective groups, and then further compared and analyzed them. In SG, we found that the body weight (P = 0.012), BMI (P < 0.001), CC (P = 0.036), AC (P = 0.017), and HC (P = 0.001) of the effective group were substantially lower than those of the ineffective group. Surprisingly, hsa-miR-6729-5p (P = 0.025), hsa-miR-4659b-5p (P = 0.024), and hsa-miR-2277-5p (P = 0.037) in the effective group were substantially higher than those in the ineffective group, and these differential known miRNAs from SG were significantly down-regulated at 1 month after surgery. In RYGB, the characteristic differences between the effective and ineffective groups were mainly manifested in body weight (P = 0.014), CC (P = 0.011), AC (P = 0.037) and FBG (P = 0.036). The expression level of hsa-miR-4662a-5p, as an up-regulated miRNA from RYGB, showed that the effective group was lower than the ineffective group in RYGB (P = 0.024). The relationships between hsa-miR-6729-5p, hsa-miR-4659b-5p, hsa-miR-2277-5p in SG or hsa-miR-4662a-5p in RYGB with clinical features were shown in Figure S2. The former was positively correlated with clinical characteristics, whereas the latter was negatively correlated. These results suggested that hsa-miR-2277-5p and hsa-miR-6729-5p might predict poor glucose metabolism in the early postoperative period after SG, whereas hsa-miR-4662a-5p was associated with improved insulin resistance after RYGB.

**Table 2:** Preoperative clinical characteristics and miRNAs expression in patients with effective or ineffective weight loss after bariatric surgery

Characteristics	SG			RYGB		
	Effective **(n = 13)**	Ineffective **(n = 5)**	P-value	Effective **(n = 6)**	Ineffective (n = 9)	P-value
Age (years)	31.62 ± 7.67	27.60 ± 4.51	0.293	34.00 ± 8.46	31.89 ± 8.08	0.635
Gender (M/F)	M (3); F (10)	M (1); F (4)	1.000	M (2); F (4)	M (6); F (3)	0.460
Weight (kg)	97.59 ± 13.14	121.72 ± 23.22	**0.012**	104.45 ± 27.24	138.06 ± 18.90	**0.014**
BMI (kg/m^2^)	35.86 ± 3.29	43.56 ± 2.43	**< 0.001**	38.91 ± 11.14	48.62 ± 4.67	0.088
NC (cm)	41.02 ± 2.82	43.40 ± 2.68	0.123	42.42 ± 6.06	47.56 ± 2.96	0.098
CC (cm)	116.04 ± 9.17	126.90 ± 8.66	**0.036**	121.25 ± 13.22	138.00 ± 8.81	**0.011**
AC (cm)	110.69 ± 10.18	125.02 ± 10.28	**0.017**	118.75 ± 20.94	142.74 ± 7.61	**0.037**
HC (cm)	116.41 ± 6.56	131.36 ± 8.89	**0.001**	120.05 ± 17.81	138.39 ± 14.98	0.050
WHR (ratio)	0.95 ± 0.08	0.95 ± 0.05	0.993	0.99 ± 0.06	1.04 ± 0.08	0.195
TCHOL (mmol/L)	5.46 ± 0.82	5.05 ± 0.79	0.348	5.81 ± 0.95	5.18 ± 0.91	0.217
HDL-C (mmol/L)	1.12 ± 0.21	1.02 ± 0.18	0.377	1.16 ± 0.22	0.99 ± 0.15	0.087
LDL-C (mmol/L)	3.28 ± 0.62	3.14 ± 0.55	0.670	3.53 ± 0.60	3.11 ± 0.67	0.229
TG (mmol/L)	1.94 ± 1.54	2.22 ± 0.86	0.712	1.49 ± 0.88	1.99 ± 1.54	0.484
FBG (mmol/L)	6.60 ± 3.00	8.58 ± 5.72	0.344	9.74 ± 4.45	5.97 ± 1.68	**0.036**
HbA1c (%)	6.52 ± 1.98	6.98 ± 2.56	0.686	8.10 ± 2.53	6.28 ± 0.80	0.142
GSP (µmol/L)	169.89 ± 71.85	172.36 ± 65.07	0.948	233.25 ± 112.51	141.49 ± 32.64	0.104
Insulin (mIU/L)	21.46 ± 13.98	28.57 ± 12.44	0.335	20.33 ± 10.92	29.39 ± 13.72	0.199
HOMA-IR	6.61 ± 5.34	10.75 ± 6.92	0.192	8.40 ± 4.75	7.50 ± 3.30	0.672
hsa-miR-6729-5p	0.82 ± 1.16	0	**0.025**	–	–	–
hsa-miR-4659b-5p	1.07 ± 1.49	0	**0.024**	–	–	–
hsa-miR-2277-5p	0.81 ± 1.24	0	**0.037**	–	–	–
hsa-miR-4662a-5p	–	–	–	0.27 ± 0.67	2.96 ± 2.87	**0.024**
has-miR-493-5p	6.79 ± 5.82	4.23 ± 4.08	0.384	6.65 ± 7.18	3.53 ± 3.53	0.279
has-miR-9-5p	67.71 ± 75.06	22.01 ± 12.61	0.202	22.79 ± 20.04	149.67 ± 226.97	0.133
has-miR-3199	1.86 ± 2.76	1.97 ± 2.72	0.938	2.64 ± 4.10	3.04 ± 3.73	0.848
has-miR-184	0.27 ± 0.99	2.95 ± 3.20	0.135	1.07 ± 2.24	76.61 ± 229.82	0.441

Data were shown as mean ± standard deviation. SG (sleeve gastrectomy); RYGB (Roux-en-Y gastric bypass); M/F (male/ female); BMI (body mass index); NC (neck circumference); CC (chest circumference); AC (abdominal circumference); HC (hip circumference); WHR (waist to hip ratio); TCHOL (total cholesterol); HDL-C (high-density lipoprotein cholesterol); LDL-C (low-density lipoprotein cholesterol); TG (triglycerides); FBG (fasting blood glucose); HbA1c (glycated hemoglobin); GSP (glycated serum protein); HOMA-IR (homeostasis model assessment of insulin resistance). %TWL (percentage total weight loss); %EWL (percentage excess weight loss). P-values < 0.05 were bolded. Hsa-miR-6729-5p, hsa-miR-4659b-5p, and hsa-miR-2277-5p were derived from differential expression of known miRNAs in SG. Has-miR-184 was derived from the differential expressions of known miRNAs in RYGB

It is worth pointing out that among the differential expressed known miRNAs in the two procedures in Fig. 4, the four miRNAs with intersection did not differ significantly in expression between the effective and ineffective groups. Furthermore, we examined the clinical change characteristics of the effective and ineffective groups before and after the two surgical strategies, respectively (Tables S3 and S4). We discovered that the effective group improved more indicators than the ineffective group for both SG and RYGB.

## Discussion

The obesity epidemic is a critical health and economic problem throughout the world. Bariatric surgery is a long-lasting, safe, and successful treatment [27]. Being the most common procedure for weight loss, SG is a restrictive weight loss surgery technique that limits the amount of food eaten at a time, altering hormones secreted by the stomach and intestines, thereby reducing hunger [28]. Patients who apply SG can reduce their body weight by more than 20% in a year [29]. RYGB includes gastrointestinal remodeling and gastric volume restriction, effectively improving lipid and glucose homeostasis, insulin sensitivity, and energy expenditure [30]. In terms of maintaining long-term weight loss and reducing the symptoms of obesity-related disorders such as diabetes, hypertension, and dyslipidemia, SG and RYGB are fairly comparable [8, 31]. Metabolic improvements appeared as early as one week to one month after surgery, although there was little weight change at this point, suggesting that other factors driving metabolic improvements after bariatric surgery were also involved [32, 33]. Our study showed that one month after SG or RYGB, both anthropometric measures and glucose metabolism measures were significantly improved, which might be the result of hormonal changes and caloric restriction. It was worth mentioning that the alleviation of dyslipidemia in SG was not as significant as that of RYGB, indicating that RYGB was more conducive to the improvement of lipid metabolism, which was similar to the conclusions of Jianfeng Dai [34]. Marianela Ackerman et al. [35] also demonstrated that RYBG was more effective in lowering LDL-C and TCHOL levels compared to SG, suggesting that RYGB gives patients additional benefits related to lipid profile.

Given the potential association of miRNAs in obesity and bariatric surgery [21], we analyzed the early differentially expressed miRNAs before and after SG or RYGB. We had found some significantly altered known and novel miRNAs before and one month after SG or RYGB. In the GO enrichment analysis, the GO terms of target genes which were predicted by the up-regulated or down-regulated miRNAs in the three taxonomic annotations appeared to be similar in the two procedures. In KEGG pathway enrichment analysis, we found some pathways related to obesity or obesity-related complications, such as MAPK signaling pathway, PI3K-Akt signaling pathway, cAMP signaling pathway, mTOR signaling pathway, metabolic pathways, insulin signaling pathway, glycerophospholipid metabolism, fatty acid biosynthesis, fructose and mannose metabolism, type II diabetes mellitus, etc. MAPK signaling pathway plays a complex and important regulatory role in adipocyte differentiation [36]. Zhinan Yin et al. [37] found that activation of the p38 MAPK-PGC-1α pathway could activate the expression of uncoupling proteins, thereby increasing the thermogenesis of brown fat to combat metabolic disorders. In bariatric surgery, activation of the PI3K-Akt signaling pathway alleviates metabolic abnormalities by reducing adipose tissue accumulation [38], and it plays an important function in controlling glucose homeostasis and insulin sensitivity [39]. The cAMP signaling pathway and mTOR signaling pathway are relatively pervasive second messengers that are significantly altered after bariatric surgery, and they are involved in many molecular pathways in obesity and metabolic diseases [40, 41]. Changes in these pathways might be associated with significant short-term improvements in metabolism following bariatric surgery. Notably, some of the identified miRNAs might still be working for the regulation of stress state, and tissue recovery after bariatric surgery. Although genes of metabolic pathways showed enrichment in the target analysis, genes involved in tissue reconstruction and healing were equally enriched, which explained to some extent the appearance of enrichment in some non-obesity and non-metabolic disease pathways such as tumor-related, chemokines, and inflammatory factors.

To screen similar differentially expressed miRNAs in SG and RYGB, we characterized the expression of known miRNAs in both procedures. In the two procedures, known miRNAs were both up-regulated for hsa-miR-493-5p, and down-regulated for hsa-miR-3199 and hsa-miR-184. These miRNAs were mainly active in tumor-related research [42–44]. Nevertheless, in our study, hsa-miR-493-5p was negatively correlated with anthropometric indicators, and glucose metabolism-related indicators, while hsa-miR-184 was positively correlated with WHR, and these results suggested possible objectives of miRNAs that underwent similar changes in SG and RYGB. Hsa-miR-9-5p was upregulated in SG but downregulated in RYGB. One study showed that hsa-miR-9-5p was higher in type II diabetes mellitus patients and was positively correlated with BMI [45].

The most widely used prognostic markers in the literature on bariatric surgery to assess the effectiveness of weight loss are the %TWL and %EWL [46]. The %EWL reports the percentage of weight loss relative to ideal BMI, and many studies defined effective weight loss as %EWL less than 50% [47, 48]. However, %EWL mainly evaluates the effect of long-term weight loss in these studies, and it is obviously inappropriate to require short-term weight loss to reach a %EWL of 50%. Some studies have defined a short-term %EWL > 25% as effective weight loss [25, 26, 49]. We set the cut-off point of %EWL at 25%, and then divided them into the weight loss effective group and the weight loss ineffective group accordingly. In SG, the effective group exhibited lower body weight, BMI, CC, AC, and HC. While in RYGB, the effective group showed lower body weight, CC, AC, and higher FBG. Muhammad A Jawad et al. found in a multicenter study that the low BMI group had higher %TWL at 6, 12 and 24 months than the high BMI group [50]. Dajin Zou et al.’s retrospective analysis of 254 patients with obesity found that the mean %EWL of the low BMI group was higher than that of the high BMI group at each time point of follow-up, and that the low BMI group had a lower reoperation rate than the high BMI group [51]. These results were consistent with our conclusion that lower anthropometric measures might be linked to more effective weight loss. The two procedures in this study had different weight loss efficiency rates, which might be a result of the short follow-up period and inconsistent preoperative clinical characteristics of the patients and should not be a focus of attention.

At the same time, we found that the expression levels of hsa-miR-6729-5p, hsa-miR-4659b-5p, and hsa-miR-2277-5p in the effective group of SG were significantly higher than those in the ineffective group. These miRNAs showed a trend of down-regulation after bariatric surgery, at least two-fold (Log_2_ FC of − 2.02, − 1.53, and − 1.16, respectively), suggesting that their presence before SG was an effective predictor of weight loss. In RYGB, only one known miRNA hsa-miR-4662a-5p was found to be different between the effective and ineffective groups, indicating that its high expression before RYGB might lead to weight loss failure. The four known miRNAs with intersections in the two procedures appeared to be poorly related to weight loss effectiveness. Additional proof is required to support the accuracy of miRNAs in predicting the effectiveness of weight loss.

## Conclusions

In general, we identified differentially expressed miRNAs preoperatively and 1 month postoperatively in SG and RYGB. The relevance of these miRNAs and their target genes to improve metabolism and reduce weight in the early stage were further analyzed. Hsa-miR-493-5p, hsa-miR-184, and hsa-miR-3199 exhibited similar changes in SG and RYGB, and the former two were correlated with clinical characteristics. Finally, %EWL > 25% was used as an evaluation index to evaluate the effectiveness of early weight loss, and related clinical characteristics and differentially expressed miRNAs were found. Hsa-miR-6729-5p, hsa-miR-4659b-5p, and hsa-miR-2277-5p were correlated with the weight loss effectiveness of SG, while hsa-miR-4662a-5p was correlated with the weight loss effectiveness of RYGB. Nevertheless, there weren’t quite as many patients in this study as there should have been, and the specific regulatory mechanism of differentially expressed miRNAs had not been further verified, which was what we need to further study in the future.

### Electronic supplementary material

Below is the link to the electronic supplementary material.

Supplementary Material 1

## Data Availability

The datasets used and analyzed during the current study are available from the corresponding authors upon reasonable request.
