# Supplementary material for: Early changes of microRNAs in blood one month after bariatric surgery
Source: Diabetol Metab Syndr. 2024 Jul 15;16:163. doi: 10.1186/s13098-024-01364-2 (PMC11251336; doi:10.1186/s13098-024-01364-2)
Supplement: Supplementary file 1 — Supplementary Material 1 [file 13098_2024_1364_MOESM1_ESM.docx]

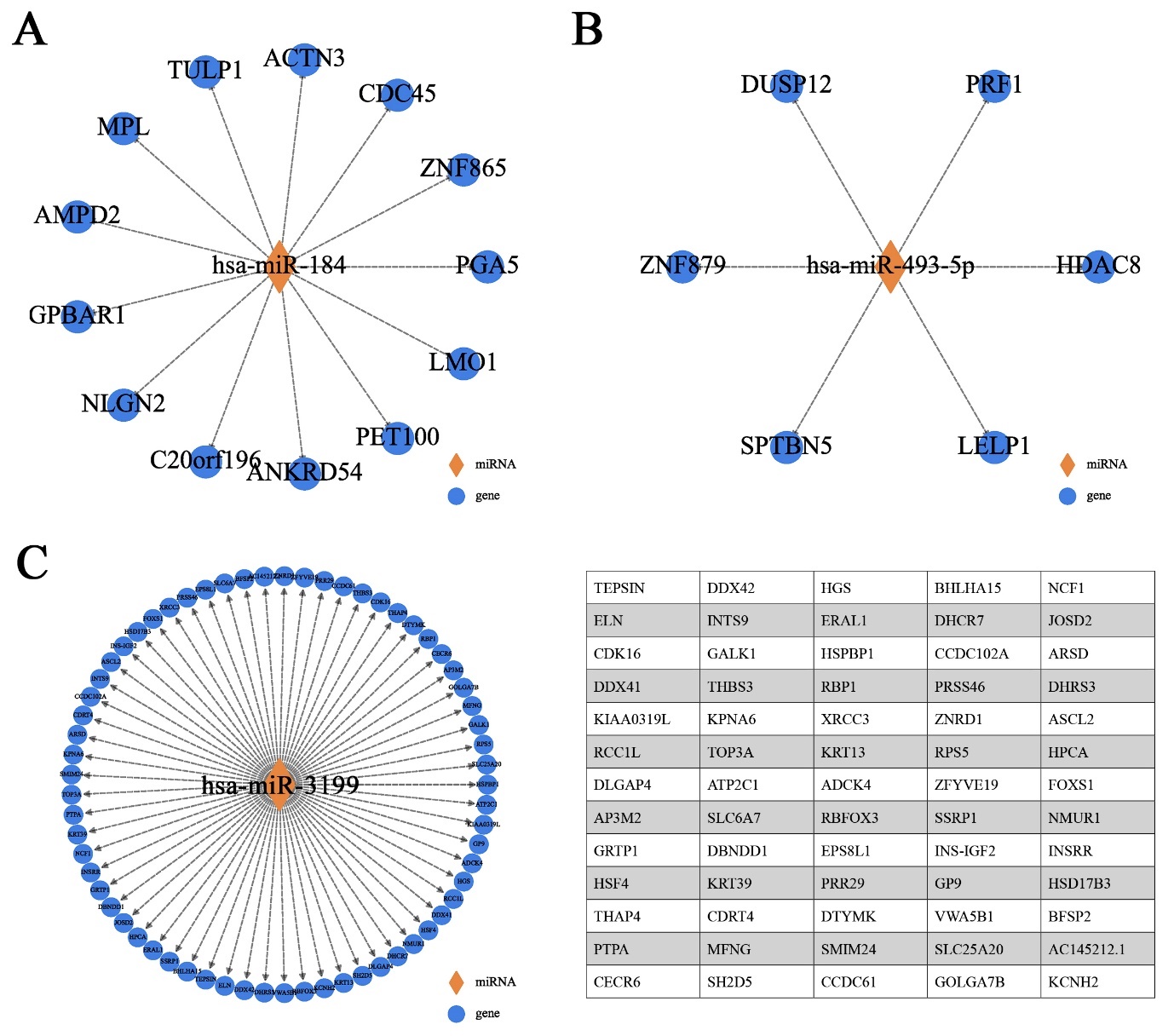


Figure S1. The co-occurrence networks of the three known miRNAs and target genes co-altered in SG and RYGB. A: The hsa-miR-184-target genes network. B: The hsa-miR-493-5P-target genes network. C: The hsa-miR-3199-target genes network. The table on the right demonstrated the target genes of hsa-miR-3199.


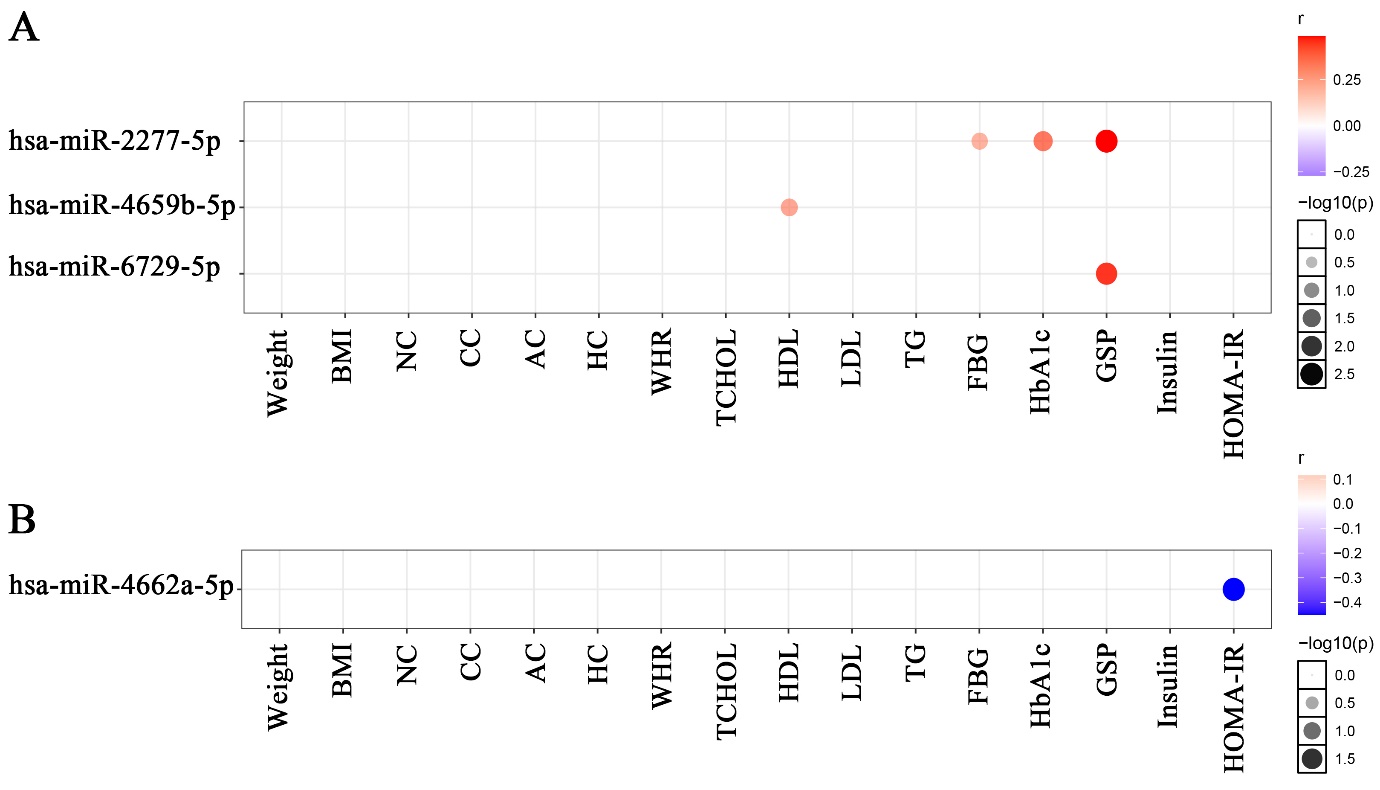


Figure S2. Pearson correlation of the known miRNAs with clinical characteristics. A: Association of differentially expressed known miRNAs that were relevant to effective weight loss in SG with clinical characteristics. B: Association of differentially expressed known miRNAs that were relevant to effective weight loss in RYGB with clinical characteristics. Only values with a P-value < 0.05 were shown.

Table S1. Changing profiles of differential expression of miRNAs for SG.

| Up-regulated miRNAs | Log_2_ FC | P-value | Down-regulated miRNAs | Log_2_ FC | P-value |
| --- | --- | --- | --- | --- | --- |
| hsa-miR-1-3p | 1.001 | 0.015 | hsa-miR-29a-5p | -1.100 | 0.022 |
| hsa-miR-493-5p | 1.081 | 0.013 | hsa-miR-548k | -1.142 | 0.038 |
| hsa-miR-411-5p | 1.134 | 0.016 | hsa-miR-2277-5p | -1.163 | 0.033 |
| hsa-miR-9-3p | 1.158 | 0.043 | hsa-miR-7845-5p | -1.173 | 0.027 |
| hsa-miR-6802-5p | 1.289 | 0.031 | hsa-miR-16-1-3p | -1.373 | 0.012 |
| hsa-miR-6823-5p | 1.296 | 0.031 | hsa-miR-519c-5p | -1.379 | 0.027 |
| hsa-miR-1229-3p | 1.296 | 0.043 | hsa-miR-518e-5p | -1.379 | 0.027 |
| hsa-miR-766-5p | 1.310 | 0.014 | hsa-miR-519b-5p | -1.379 | 0.027 |
| hsa-miR-6721-5p | 1.326 | 0.024 | hsa-miR-522-5p | -1.379 | 0.027 |
| hsa-miR-4738-3p | 1.330 | 0.031 | hsa-miR-523-5p | -1.379 | 0.027 |
| hsa-miR-219a-2-3p | 1.347 | 0.031 | hsa-miR-520h | -1.410 | 0.027 |
| hsa-miR-9-5p | 1.386 | 0.021 | hsa-miR-125b-1-3p | -1.439 | 0.039 |
| hsa-miR-377-5p | 1.451 | 0.028 | hsa-miR-3944-5p | -1.443 | 0.027 |
| hsa-miR-34b-3p | 1.499 | 0.023 | novel_38 | -1.448 | 0.013 |
| hsa-miR-5581-3p | 1.500 | 0.013 | novel_34 | -1.464 | 0.027 |
| hsa-miR-6859-5p | 1.501 | 0.048 | hsa-miR-5584-5p | -1.479 | 0.027 |
| hsa-miR-548ag | 1.609 | 0.028 | hsa-miR-624-3p | -1.500 | 0.027 |
| hsa-miR-7848-3p | 1.617 | 0.013 | hsa-miR-4637 | -1.504 | 0.013 |
| hsa-miR-4716-3p | 1.722 | 0.005 | hsa-miR-942-3p | -1.511 | 0.027 |
| novel_242* | 2.235 | 0.004 | hsa-miR-4659b-5p | -1.526 | 0.024 |
| novel_71 | 2.658 | 0.021 | hsa-miR-184 | -1.559 | 0.042 |
| novel_224 | 3.108 | 0.003 | hsa-miR-3199 | -1.581 | 0.021 |
| novel_221 | 3.475 | 0.001 | hsa-miR-3657 | -1.614 | 0.013 |
| novel_161 | 3.607 | < 0.001 | novel_97 | -1.682 | 0.045 |
| novel_165 | 3.782 | 0.001 | novel_25 | -1.720 | 0.041 |
| novel_140 | 3.813 | 0.002 | hsa-miR-6754-3p | -1.773 | 0.007 |
| novel_146 | 3.916 | 0.002 | hsa-miR-515-5p | -1.803 | 0.043 |
| novel_322 | 4.135 | 0.001 | hsa-miR-4646-5p | -1.836 | 0.007 |
| novel_244 | 4.140 | 0.002 | hsa-let-7f-2-3p | -1.840 | 0.007 |
| novel_168 | 4.542 | < 0.001 | hsa-miR-3912-3p | -1.882 | 0.002 |
| novel_212 | 6.075 | 0.002 | hsa-miR-203a-3p | -1.895 | 0.021 |
|  |  |  | novel_19 | -2.006 | 0.035 |
|  |  |  | hsa-miR-6729-5p | -2.025 | 0.002 |
|  |  |  | hsa-miR-517c-3p | -2.029 | 0.004 |
|  |  |  | novel_272 | -2.058 | 0.001 |
|  |  |  | novel_35 | -2.171 | 0.005 |
|  |  |  | novel_78 | -2.244 | 0.006 |
|  |  |  | novel_197 | -2.502 | 0.002 |
|  |  |  | novel_268 | -2.525 | 0.001 |
|  |  |  | novel_7 | -2.553 | 0.005 |
|  |  |  | novel_94 | -2.649 | 0.033 |
|  |  |  | novel_5 | -2.690 | 0.006 |
|  |  |  | hsa-miR-10401-3p | -2.712 | < 0.001 |
|  |  |  | novel_123 | -2.725 | < 0.001 |
|  |  |  | novel_108 | -3.188 | 0.001 |
|  |  |  | hsa-miR-6859-3p | -3.607 | < 0.001 |
|  |  |  | novel_43 | -3.637 | 0.002 |
|  |  |  | novel_104 | -3.705 | 0.001 |
|  |  |  | novel_109 | -3.812 | 0.004 |
|  |  |  | novel_129 | -4.180 | 0.003 |
|  |  |  | novel_65 | -4.275 | 0.004 |
|  |  |  | novel_276 | -4.539 | 0.003 |
|  |  |  | novel_41 | -4.774 | 0.002 |
|  |  |  | novel_76 | -6.362 | 0.002 |

* We integrated miREvo and mirdeep2, the miRNAs prediction softwares, to perform the analysis of novel miRNAs. All sequences are available upon request from the corresponding authors. The data was ordered by Log_2_ FC.

Table S2. Changing profiles of differential expression of miRNAs for RYGB.

| Up-regulated miRNAs | Log_2_ FC | P-value | Down-regulated miRNAs | Log_2_ FC | P-value |
| --- | --- | --- | --- | --- | --- |
| hsa-miR-493-5p | 1.082 | 0.024 | hsa-miR-27a-5p | -1.003 | 0.041 |
| hsa-miR-4662a-5p | 1.323 | 0.016 | hsa-miR-33b-5p | -1.057 | 0.025 |
| hsa-miR-122-3p | 1.396 | 0.040 | hsa-miR-1307-5p | -1.173 | 0.002 |
| hsa-miR-202-3p | 1.476 | 0.032 | hsa-miR-5006-5p | -1.243 | 0.045 |
| hsa-miR-4677-5p | 1.482 | 0.033 | hsa-miR-551b-5p | -1.291 | 0.045 |
| hsa-miR-30b-3p | 1.632 | 0.030 | hsa-miR-524-5p | -1.297 | 0.045 |
| hsa-miR-873-3p | 1.660 | 0.035 | hsa-miR-3620-3p | -1.299 | 0.045 |
| novel_175* | 1.704 | 0.019 | hsa-miR-1246 | -1.311 | 0.045 |
| hsa-miR-4781-5p | 1.763 | 0.012 | hsa-miR-6806-3p | -1.329 | 0.045 |
| hsa-miR-708-3p | 1.770 | 0.033 | hsa-miR-1256 | -1.344 | 0.045 |
| novel_88 | 1.823 | 0.033 | hsa-miR-320e | -1.367 | 0.045 |
| hsa-miR-130a-5p | 1.830 | 0.019 | hsa-miR-6881-3p | -1.379 | 0.045 |
| novel_105 | 1.834 | 0.033 | hsa-miR-96-3p | -1.387 | 0.045 |
| hsa-miR-7110-3p | 1.898 | 0.019 | hsa-miR-490-5p | -1.410 | 0.029 |
| hsa-miR-6791-3p | 1.925 | 0.019 | hsa-miR-3176 | -1.448 | 0.036 |
| hsa-miR-122b-5p | 1.973 | 0.012 | hsa-miR-219a-5p | -1.497 | 0.045 |
| hsa-miR-487a-5p | 2.004 | 0.012 | hsa-miR-4687-5p | -1.584 | 0.047 |
| hsa-miR-6516-3p | 2.011 | 0.012 | hsa-miR-6791-5p | -1.611 | 0.014 |
| novel_100 | 2.128 | 0.010 | hsa-miR-487a-3p | -1.630 | 0.014 |
| hsa-miR-3130-5p | 2.190 | 0.007 | hsa-miR-200a-5p | -1.649 | 0.014 |
| hsa-miR-4632-3p | 2.191 | 0.004 | hsa-miR-580-3p | -1.710 | 0.012 |
| hsa-miR-4536-5p | 2.242 | 0.008 | hsa-miR-152-5p | -1.807 | 0.014 |
| hsa-miR-3202 | 2.323 | 0.007 | hsa-miR-3188 | -1.889 | 0.005 |
| hsa-miR-1976 | 2.381 | 0.003 | hsa-miR-9-5p | -1.921 | 0.004 |
| novel_48 | 2.531 | 0.015 | hsa-miR-7847-3p | -1.926 | 0.005 |
| novel_192 | 2.721 | 0.006 | novel_71 | -2.449 | 0.049 |
| novel_158 | 2.762 | 0.009 | hsa-miR-3199 | -2.596 | 0.001 |
| novel_197 | 3.159 | 0.003 | novel_140 | -2.688 | 0.011 |
| novel_123 | 3.359 | 0.010 | novel_68 | -2.703 | 0.017 |
| novel_153 | 3.381 | 0.011 | novel_279 | -3.195 | 0.002 |
| novel_101 | 3.452 | 0.004 | hsa-miR-124-3p | -3.361 | 0.003 |
| novel_199 | 3.675 | 0.013 | novel_339 | -3.690 | 0.001 |
| novel_164 | 3.685 | 0.012 | novel_52 | -3.757 | 0.006 |
| novel_267 | 3.919 | 0.016 | novel_217 | -4.366 | 0.002 |
| novel_168 | 3.920 | 0.008 | novel_328 | -4.551 | 0.001 |
| novel_109 | 4.084 | 0.012 | novel_299 | -4.989 | 0.001 |
| novel_159 | 4.529 | 0.016 | novel_69 | -6.125 | 0.003 |
|  |  |  | hsa-miR-184 | -6.988 | < 0.001 |
|  |  |  | novel_76 | -7.468 | 0.002 |

* We integrated miREvo and mirdeep2, the miRNAs prediction softwares, to perform the analysis of novel miRNAs. All sequences are available upon request from the corresponding authors. The data was ordered by Log_2_ FC.

Table S3. Clinical characteristics of effective or ineffective weight loss in patients undergoing SG preoperatively and at 1 month postoperatively.

| Characteristics | Effective (n = 13) | | |  | Ineffective (n = 5) | | |
| --- | --- | --- | --- | --- | --- | --- | --- |
|  | **Preoperative** | **Postoperative** | **P-value** |  | **Preoperative** | **Postoperative** | **P-value** |
| Age (years) | 31.62 ± 7.67 | – | – |  | 27.60 ± 4.51 | – | – |
| Gender (M/F) | M (3); F (10) | – | – |  | M (1); F (4) | – | – |
| Weight (kg) | 97.59 ± 13.14 | 86.64 ± 12.37 | **< 0.001** |  | 121.72 ± 23.22 | 111.5 ± 20.19 | **0.002** |
| BMI (kg/m^2^) | 35.86 ± 3.29 | 31.65 ± 3.15 | **< 0.001** |  | 43.56 ± 2.43 | 39.34 ± 1.87 | **0.001** |
| NC (cm) | 41.02 ± 2.82 | 38.7 ± 2.64 | **< 0.001** |  | 43.38 ± 3.09 | 41.20 ± 3.70 | **0.049** |
| CC (cm) | 116.04 ± 9.17 | 109.58 ± 7.31 | **< 0.001** |  | 129.5 ± 7.40 | 119.53 ± 9.28 | **0.009** |
| AC (cm) | 110.69 ± 10.18 | 103.83 ± 10.52 | **0.002** |  | 126.03 ± 11.58 | 115.98 ± 12.23 | **0.002** |
| HC (cm) | 116.41 ± 6.56 | 110.73 ± 7.08 | **< 0.001** |  | 130.33 ± 9.91 | 123.13 ± 6.01 | 0.295 |
| WHR (ratio) | 0.95 ± 0.08 | 0.94 ± 0.06 | 0.330 |  | 0.97 ± 0.05 | 0.94 ± 0.08 | 0.572 |
| TCHOL (mmol/L) | 5.46 ± 0.82 | 5.11 ± 1.02 | 0.284 |  | 5.05 ± 0.79 | 4.79 ± 0.64 | 0.474 |
| HDL-C (mmol/L) | 1.12 ± 0.21 | 1.04 ± 0.17 | 0.067 |  | 1.02 ± 0.18 | 1.01 ± 0.18 | 0.881 |
| LDL-C (mmol/L) | 3.28 ± 0.62 | 3.09 ± 0.84 | 0.446 |  | 3.14 ± 0.55 | 2.77 ± 0.35 | 0.309 |
| TG (mmol/L) | 1.94 ± 1.54 | 1.20 ± 0.41 | 0.070 |  | 2.22 ± 0.86 | 1.66 ± 0.73 | 0.074 |
| FBG (mmol/L) | 6.60 ± 3.00 | 4.98 ± 0.58 | 0.062 |  | 8.58 ± 5.72 | 5.08 ± 1.12 | 0.176 |
| HbA1c (%) | 6.52 ± 1.98 | 5.79 ± 1.17 | **0.012** |  | 6.98 ± 2.56 | 5.96 ± 1.40 | 0.144 |
| GSP (µmol/L) | 169.89 ± 71.85 | 139.15 ± 19.07 | 0.077 |  | 172.36 ± 65.07 | 129.20 ± 21.21 | 0.141 |
| Insulin (mIU/L) | 21.46 ± 13.98 | 9.61 ± 5.77 | **0.014** |  | 28.57 ± 12.44 | 16.14 ± 8.34 | 0.127 |
| HOMA-IR | 6.61 ± 5.34 | 2.20 ± 1.47 | **0.011** |  | 10.75 ± 6.92 | 3.93 ± 2.94 | **0.033** |

Data were shown as mean ± standard deviation. P-values < 0.05 were bolded.

Table S4. Clinical characteristics of effective or ineffective weight loss in patients undergoing RYGB preoperatively and at 1 month postoperatively.

| Characteristics | Effective (n = 6) | | |  | Ineffective (n = 9) | | |
| --- | --- | --- | --- | --- | --- | --- | --- |
|  | **Preoperative** | **Postoperative** | **P-value** |  | **Preoperative** | **Postoperative** | **P-value** |
| Age (years) | 34.00 ± 8.46 | – | – |  | 31.89 ± 8.08 | – | – |
| Gender (M/F) | M (2); F (4) | – | – |  | M (6); F (3) | – | – |
| Weight (kg) | 104.45 ± 27.24 | 91.60 ± 24.52 | **< 0.001** |  | 138.06 ± 18.90 | 124.52 ± 17.03 | **< 0.001** |
| BMI (kg/m2) | 38.91 ± 11.14 | 33.73 ± 9.50 | **0.001** |  | 48.62 ± 4.67 | 43.43 ± 3.85 | **< 0.001** |
| NC (cm) | 42.42 ± 6.06 | 39.80 ± 5.09 | **0.005** |  | 47.56 ± 2.96 | 45.76 ± 3.29 | **0.008** |
| CC (cm) | 121.25 ± 13.22 | 114.00 ± 11.61 | **0.005** |  | 138.00 ± 8.81 | 129.33 ± 7.98 | **0.002** |
| AC (cm) | 118.75 ± 20.94 | 110.67 ± 21.03 | **< 0.001** |  | 142.74 ± 7.61 | 131.39 ± 8.72 | **< 0.001** |
| HC (cm) | 120.05 ± 17.81 | 112.92 ± 16.31 | **0.015** |  | 138.39 ± 14.98 | 136.17 ± 13.17 | 0.413 |
| WHR (ratio) | 0.99 ± 0.06 | 0.98 ± 0.08 | 0.595 |  | 1.04 ± 0.08 | 0.97 ± 0.05 | **0.033** |
| TCHOL (mmol/L) | 5.81 ± 0.95 | 4.70 ± 0.61 | **0.043** |  | 5.18 ± 0.91 | 4.53 ± 0.80 | **0.020** |
| HDL-C (mmol/L) | 1.16 ± 0.22 | 0.92 ± 0.19 | **0.015** |  | 0.99 ± 0.15 | 0.89 ± 0.09 | 0.089 |
| LDL-C (mmol/L) | 3.53 ± 0.60 | 2.89 ± 0.55 | **0.031** |  | 3.11 ± 0.67 | 2.72 ± 0.63 | 0.080 |
| TG (mmol/L) | 1.49 ± 0.88 | 1.30 ± 0.44 | 0.619 |  | 1.99 ± 1.54 | 1.64 ± 0.82 | 0.208 |
| FBG (mmol/L) | 9.74 ± 4.45 | 5.82 ± 1.13 | 0.059 |  | 5.97 ± 1.68 | 5.39 ± 1.08 | 0.117 |
| HbA1c (%) | 8.10 ± 2.53 | 6.48 ± 1.68 | **0.011** |  | 6.28 ± 0.80 | 5.64 ± 0.78 | **< 0.001** |
| GSP (µmol/L) | 233.25 ± 112.51 | 156.83 ± 44.79 | 0.059 |  | 141.49 ± 32.64 | 123.44 ± 15.44 | 0.062 |
| Insulin (mIU/L) | 20.33 ± 10.92 | 9.30 ± 4.71 | **0.046** |  | 29.39 ± 13.72 | 16.78 ± 5.51 | **0.013** |
| HOMA-IR | 8.40 ± 4.75 | 2.28 ± 1.02 | **0.027** |  | 7.50 ± 3.30 | 3.96 ± 1.34 | **0.011** |

Data were shown as mean ± standard deviation. P-values < 0.05 were bolded.
